# Traumatic optic neuropathy management: a systematic review

**DOI:** 10.1038/s41433-024-03129-7

**Published:** 2024-06-11

**Authors:** Richard J. Blanch, Iric John Joseph, Kimberly Cockerham

**Affiliations:** 1 Royal Centre for Defence Medicine, Birmingham, UK; 2 Institute of Inflammation and Ageing, University of Birmingham, Birmingham, UK; 3 Birmingham Neuro-Ophthalmology, University Hospitals Birmingham NHS Foundation Trust, Birmingham, UK; 4 Oculoplastics- Orbit-Neuro-Ophthalmology, Senta Clinic, San Diego, CA USA

**Keywords:** Outcomes research, Brain injuries, Optic nerve diseases

## Abstract

**Background:**

Traumatic optic neuropathy is classically described in up to 8% of patients with traumatic brain injury (TBI), but subclinical or undiagnosed optic nerve damage is much more common. When more sensitive testing is performed, at least half of patients with moderate to severe TBI demonstrate visual field defects or optic atrophy on examination with optical coherence tomography. Acute optic nerve compression and ischaemia in orbital compartment syndrome require urgent surgical and medical intervention to lower the intraocular pressure and diminish the risk of permanent optic nerve dysfunction. Other manifestations of traumatic optic neuropathy have more variable treatments in international practice.

**Methods:**

We conducted a systematic review of traumatic optic neuropathy treatments in accordance with the PRISMA (Preferred Reporting Items for Systematic Reviews and Meta-Analyses) statement.

**Results:**

We included three randomised controlled trials of intravenous methylprednisolone (IVMP), erythropoietin, and levodopa-carbidopa combination, with no evidence of benefit for any treatment. In addition, large studies in TBI have found strong evidence of increased mortality in patients treated with megadose IVMP.

**Conclusions:**

There is therefore no evidence of benefit for any medical treatment and strong evidence of harm from IVMP. There is also no evidence of benefit for optic canal decompression for traumatic optic neuropathy. Orbital compartment syndrome is a separate entity that requires both medical and surgical interventions to prevent visual loss.

## Abstract (zh)

背景: 在创伤性脑损伤 (TBI) 患者中, 有高达8%的患者有典型的创伤性视神经损伤, 但亚临床或未确诊的视神经损伤更为常见。在进行更灵敏的检查时, 至少有一半中到重度脑外伤患者在OCT检查中出视野缺陷或视神经萎缩。眼眶间隔综合征的急性视神经压迫和缺血需要紧急手术和药物干预, 以降低眼压和减少永久性视神经功能障碍的风险。国际上对于外伤性视神经病变的其他表现有不同的治疗方法。

方法: 我们根据PRISMA(系统回顾和荟萃分析的首选报告项目)声明对外伤性视神经病变的治疗进行了系统综述。

结果: 我们纳入了三组随机对照试验, 分别为静脉注射甲强龙(IVMP)、促红细胞生成素和左旋多巴-卡比多巴联合用药, 没有证据表明任何治疗是有效的。此外, 在TBI的大型研究中发现, 强有力的证据表明, 接受大剂量IVMP静脉注射治疗的患者死亡率增加。

结论: 因此, 没有强有力的证据表明IVMP对任何治疗有益或有害。也没有证据表明视神经管减压术对外伤性视神经病变有益。眼眶间隔综合征是一个同时需要内外科干预以防止视力丧失的需要考虑的独立因素。

## Traumatic brain injury (TBI)

TBI may occur after open or closed head injury and is always associated with altered brain function. Closed injury, with intact skin and skull, is most common and the mechanism of injury is acceleration and deceleration of the brain within the skull, twisting, stretching, and damaging the axons and blood vessels. Initial damage with acute injury to the axonal and nerve soma is called primary injury from the physical impact. Secondary injuries may occur from pathophysiological consequences and neurodegenerative processes [1, 2]. The biochemical reactions taking place after the primary injury and their role in initiating a cycle of neurodegeneration are poorly understood [3].

TBI may be categorised as mild, moderate, or severe, although symptom severity does not always coincide with injury severity, making TBI severity difficult to diagnose and TBI difficult to manage. Whilst there are many classification systems for TBI severity, the Mayo classification is used most frequently (Table 1).

**Table 1:** The Mayo classification of TBI [50].

Criteria	Mild	Moderate	Severe
GCS score	13–15	9–12	≤8
Post-traumatic amnesia	<24 h	24 h to 1 week	>1 week
Loss of consciousness	Up to 30 min	30 min to 24 h	>24 h
Alteration of consciousness/mental state	<24 h	>24 h, severity based on other criteria	>24 h, severity based on other criteria

Mild TBI is usually classified in terms of normal structural brain imaging on computed tomography and has common symptoms of headache, dizziness, nausea, confusion, and disorientation, which can last indefinitely [4, 5]. Moderate to severe TBI may be associated with haematomas and tissue necrosis, losing brain functionality, and releasing toxins with more prolonged unconsciousness and long-term neurological impairment [5].

## Visual loss associated with TBI

In 594 TBI patients with ophthalmic findings, 28.0–51.8% suffered eyelid ecchymosis, 38.6–44.4% subconjunctival haemorrhage, 43.1% chemosis, 41.4% lid oedema, and 22.5% a lacerating injury [6–9]. Classical traumatic optic neuropathy (TON) is reported in 0.5–8% of civilian TBI cases [10–15]. TON is more common after combat ocular trauma, occurring in up to 20%, with 40% being legally blind (20/400 or worse) as a result of the traumatic alterations [16–18].

The visual pathways are vulnerable to TBI, especially the long axons between the eye and lateral geniculate body, which may be damaged as part of diffuse axonal injury [4, 19]. TBI indicators are therefore commonly found in the retina, with 11–54% of TBI patients suffering visual field defects, and ganglion cell layer (GCL) thinning in 31–47%, which may or may not be associated with visual symptoms and is considered to be subclinical TON [20–24]. These changes, including visual field defects and classical TON, occur across the range of TBI severity from mild to severe, although are more common with increasing TBI severity [Laws and Blanch—unpublished data]. TON therefore represents a spectrum of ophthalmic TBI manifestations, including subtle chronic neurodegeneration detectable on OCT [25], subacute RNFL and GCL loss weeks to months after injury, and severe and catastrophic visual loss associated with direct or indirect injury to the anterior visual pathways.

## Acute visual loss after TBI

Patients with TBI often suffer from skull and facial fractures and may develop orbital compartment syndrome secondary to intra-orbital haematoma. The timescale within which irreversible retinal ischaemia occurs is poorly defined and varies depending on the orbital perfusion pressure (a function of orbital pressure and blood pressure), and pre-existing optic nerve and retinal health. Anecdotal reports indicate that orbital decompression should be performed within 2–4 h, although irreversible ischaemia may occur within less than 60 min [26].

Initial treatment should be immediate lateral superior and inferior canthotomy and cantholysis, and simultaneous administration of intravenous mannitol and acetazolamide if orbital or intraocular pressure remains elevated. Topical glaucoma drops to diminish production of aqueous such as timolol are often also utilised. If the orbit remains tense and retinal perfusion is impaired, further decompression may be achieved through an upper lid skin crease incision to allow orbital fat prolapse or an inferior lateral transconjunctival evacuation of the retained clot, and ultimately bony decompression through an anterior or endonasal approach to the floor or medial wall.

## Traumatic optic neuropathy treatment in international practice

In a survey of 42 major trauma centres internationally, 64% routinely administered systemic corticosteroids for TON in widely variable doses and durations (Table 2).

**Table 2:** International variation in practice of treatment for TON [36].

	*N* (%)
Routinely administered systemic corticosteroids	21 (63.6%)
IV	12 (36.4%)
Oral	8 (24.2%)
Both IV and Oral	1 (3.0%)
Systemic corticosteroid dosage	
Prednisone 1 mg/kg daily (Moderate)	7 (21.2%)
Methylprednisolone 1 g daily (High)	12 (36.4%)
Methylprednisolone >2 g daily (Mega)	1 (3.0%)
Other	1 (3.0%)
Average duration of steroid therapy (days)	15.98 ± 11.87

## Evidence for treatment of traumatic optic neuropathy

### Methods

To evaluate potential treatments for TON, we conducted a systematic review, in accordance with the PRISMA (Preferred Reporting Items for Systematic Reviews and Meta-Analyses) statement [27].

### Search strategy

Searches were carried out on 29 Dec 2023, of Pubmed, Medline, Embase, CINAHL, and Clinicaltrials.gov with the search terms (‘traumatic brain injur*’ OR ‘head injur*’ OR ‘traumatic optic neuropathy’ OR ‘craniocerebral trauma’ OR ‘TBI’) AND (‘retina’ OR ‘optic nerve’ OR ‘eye’ OR ‘ocul*’), restricted to select for randomised controlled trials as previously described [28]. Searches were imported in Rayyan, and titles and abstracts were screened by two authors (RJB and IJ) with disagreements settled by discussion [29]. If the paper could not be included or excluded with certainty on the basis of the abstract, then the full text was read. Included study reference and citation lists were examined for additional studies which may meet inclusion criteria.

### Inclusion and exclusion criteria

Randomised controlled trials evaluating any treatments for traumatic neuropathy in humans were included. This review did not include orbital compartment syndrome as it is a distinct entity with an alternate standard of care therapeutic intervention detailed above. Only studies evaluating treatments administered within the first month after injury were included. Only papers published in indexed medical journals were included. Studies without control groups (i.e. comparing two different treatments) were excluded. No restrictions were placed on language, patient age, year of publication or mechanisms of injury.

### Risk of bias assessment

Two authors independently assessed the potential risk of bias in included studies using the Cochrane Risk of Bias (RoB2) tool [30].

### Data extraction

Data were extracted in duplicate by two authors (RB and IJ). The variables recorded included: study information (first author, publication year, country of origin), participant information (number of patients in each arm, gender, patient age in each arm), study inclusion and exclusion criteria, primary and secondary outcomes, intervention, follow-up for each group, safety information.

### Statistical methods

Data were synthesised narratively without meta-analysis.

## Results

**Fig. 1:**
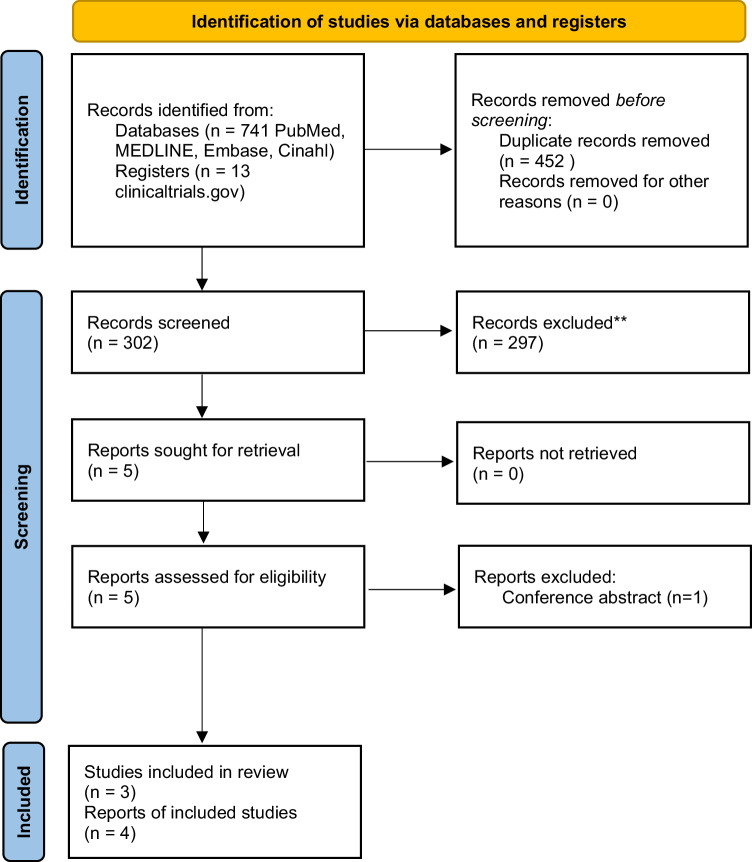
PRISMA 2020 flow diagram for new systematic reviews that included searches of databases and registers only [31].

Three studies met the inclusion criteria. A PRISMA flow diagram of the search results is presented in Fig. 1 [31].

### Characteristics of included studies

The included studies assessed three different treatments in a total of 157 included patients (across treatment and placebo arms). Patient characteristics are presented in Table 3, with studies recruiting patients with indirect TON occurring within the prior 6 days to 3 weeks.

**Table 3:** Characteristics of included patients.

First Author, year	Country	Male/ female	Age (years)	Inclusion Criteria	Exclusion Criteria	Patients recruited	Patients included in the analysis	Length of Follow-Up
Kashkouli, 2017 [32, 33]	Iran	88 male 12 female	53% <25	Indirect TON; <3 weeks prior	Other injuries affecting visual function; reduced consciousness level; glaucoma; diabetic neuropathy	117 (EPO, 85; Obs, 16; IVMP 16)	100 (EPO, 69; Obs, 15; IVMP 16)	Protocol > 3 months; mean 208 days
Razeghinejad, 2010 [34]	Iran	26 male 1 female	LD 21 ± 8.9; Pl 25 ± 6.1	Indirect TON < 6 days prior	Direct TON; ON avulsion; open globe/orbital injury; orbital/optic canal fractures	32 (LD 20, Pl 30)	26 (LD 16, Pl 10)	LD 17.5 ± 6.6 months; Pl 14.3 ± 5.3 months
Entezari, 2007 [35]	Iran	31 male	Pl 29 ± 10.2; IVMP 30.2 ± 10.6	Indirect TON < 7 days prior	Direct TON; open globe injury; other cause of reduced vision; optic nerve decompression surgery; orbital fractures	31 (IVMP 16; Pl 15)	31 (IVMP 16; Pl 15)	3 months

*EPO* erythropoietin, *IVMP* intravenous methylprednisolone, *LD* levodopa-carbidopa combination, Obs observation, *Pl* placebo, *TON* traumatic optic neuropathy.

### Risk of bias in and quality of included studies

A risk of bias analysis using the RoB2 tool is presented in Table 4. The authors assessed a high risk of bias in two out of three included studies. For the study by Kashkouli et al. [32, 33], this was principal because they did not mask study participants to treatment allocation, and, despite randomising subjects to particular study groups, allowed subjects to choose to change allocation, with most subjects choosing to be transferred to the treatment group (EPO). In addition, only 69 of 85 patients receiving EPO were analysed because 14 were lost to follow-up and 16 received non-protocol interventions [32].

**Table 4:**
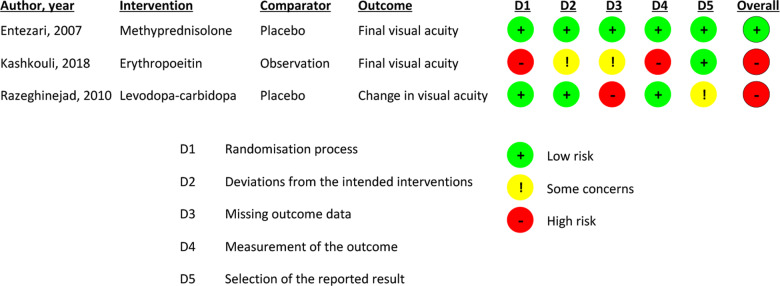
Risk of bias for included studies assessed using the Cochrane Risk of Bias (RoB2) tool [30].

Razeghinejad et al. analysed follow-up data on only 10 of 30 patients randomised to placebo and 16 of 20 patients randomised to levodopa [34]. It is therefore possible that the results could be significantly different were data available on all patients in the placebo group, and it is also possible that patients with a good visual outcome may be less likely to attend follow-up [34].

Only Kashkouli et al. reported a pre-specified power calculation, with the effect size based on a small pilot study with retrospective control group [32, 33].

### Findings

The findings of included studies are summarised in Table 5. One study (included in two reports) examined the effectiveness of erythropoietin (EPO) infusion, the smaller of the two including 100 patients and found no evidence of differential visual recovery with treatment compared to observation or intravenous methylprednisolone (IVMP) [32, 33], including 117 patients and found no evidence that EPO was associated with higher rates of visual recovery than observation or IVMP [32].

**Table 5:** Characteristics of included patients.

First Author, year	Intervention	Control groups	Primary outcome	Secondary outcomes	Safety concerns
Kashkouli, 2017 [32, 33]	EPO 20,000 IU/day for 3 days [10,000 IU/day if <13 years]	1) IVMP 250 mg QDS for 3 days 2) Observation	Improved BCVA: No evidence for a difference between groups EPO 35.78 ± 32.76%; IVMP 29.15 ± 31.71%; Obs 24.17 ± 28.46% (*p* = 0.38)	Change in colour vision: No difference between groups (*p* = 0.62) Change in RAPD: No difference between groups (*p* = 0.66)	Not reported
Razeghinejad, 2010 [34]	Levodopa 100 mg/carbidopa 10 mg TDS for 1 month PLUS IVMP 250 mg QDS for 3 days then oral prednisolone 1 mg/kg for 11 days	Placebo PLUS IVMP 250 mg QDS for 3 days then oral prednisolone 1 mg/kg for 11 days	Not specified	Change in VA: Improvement in 56% LD and 10% Pl (*p* = 0.02) Visual acuity improvement in patients with VA < HM or NLP: No difference between groups (*p* = 0.1 for <HM and *p* = 1 for NLP) Unrecordable PVEP: No difference between groups 9/16 LD, 9/10 Pl (*p* = 0.09)	Not reported
Entezari, 2007 [35]	IVMP 250 mg QDS for 3 days then oral prednisolone 1 mg/kg for 11 days	Placebo	Final BCVA: No evidence for a difference between groups 0.67 (95% CI-1.54–0.2; *p* = 0.12)	Change in BCVA: No difference between groups, OR 1.9 (95% CI 0.45–8.33; *p* = 0.38)	Not reported

*EPO* erythropoietin, *BCVA* best corrected visual acuity, *IVMP* intravenous methylprednisolone, *LD* levodopa-carbidopa combination, *Obs* observation, *Pl* placebo, *VA* visual acuity.

Two studies examined the effect of IVMP on visual function seven days to three weeks after TON, with a total of 32 patients treated with IVMP (at doses of 1 g each day for 3–11 days followed by up to 14 days of oral prednisolone and compared to a total of 30 patients treated with either observation or placebo [32, 33, 35]. No study found evidence of a beneficial effect of IVMP on visual recovery.

One study examined the effect of levodopa-carbidopa combination with IVMP 1 g each day in 20 patients compared to IVMP alone in 12 patients, but conducted a per-protocol analysis on 16 levodopa (out of 20 treated) and 10 placebo-treated (out of 30 treated) patients not lost to follow up, finding weak evidence (*p* = 0.02) that patients treated with levodopa were more likely to experience two Snellen lines of visual recovery than patients treated with IVMP alone, although this study was assessed to be at high risk of bias [34].

No studies reported safety data.

## Discussion

We included three RCTs of treatment for TON, none of which provided convincing evidence for a treatment benefit of EPO, IVMP or levodopa-carbidopa combination, either from negative results or from high risk of bias. Despite the lack of convincing RCT data for efficacy, IVMP remains in common usage internationally [36].

The CRASH study, published in 2005 recruited 10,008 patients examined within less than 8 h after TBI, randomising them to placebo or 11.6 g intravenous methylprednisolone over the first 24 h, followed by 21.2 g over the second 48 h [37]. Patients were more likely to have an outcome of death and the composite outcome of death or severe disability when treated with corticosteroids than with placebo. That detrimental difference in outcome was qualitatively greater in patients with moderate TBI than in patients with severe TBI, and with increasing time since injury [37].

In addition to the RCT included in this review, a single prospective non-randomised study of TON compared three managements: 1) corticosteroids (ranging from megadose to low dose), 2) optic canal decompression and 3) observation. Patients were most likely to recover visual function in the observation group and least likely to recover visual function in the optic canal decompression group [38]. A Cochrane review found no evidence that corticosteroid treatment was of benefit in TON [39]. Another trial that compared megadose corticosteroid treatment to surgical decompression found that there was no difference in visual recovery in the two groups, although there was no control group [40, 41]. There is therefore no evidence of benefit to treatment with systemic steroids in TON and strong evidence for increased risk of death and death or severe disability when patients are treated with high-dose corticosteroids after TBI. There is also no evidence that surgical decompression is effective. The only circumstances under which surgical decompression is recommended in the treatment of TON is therefore to relieve orbital compartment syndrome.

Other included studies looked at treatment with erythropoietin and levodopa, finding no strong evidence of benefit [32–34]. In particular, whilst the included study examining the effect of levodopa-carbidopa combination reported weak evidence of benefit, they conducted a per-protocol analysis rather than intention to treat analysis and their small patient numbers, high drop-out, and small effect size would be very sensitive to a different outcome in even one or two of the patients lost to follow up [34]. Nonetheless, the potentially positive effect of levodopa in this small study potentially justifies further testing in a well-designed RCT with a priori power calculation.

It is notable that all included studies were performed in a single country (Iran) and none reported safety data. The studies were all small and may have lacked power. TON detected by ophthalmologists in routine practice is a relatively rare condition, with one UK British Ophthalmic Surveillance Unit study reporting an annual incidence of 1.005 per million population [42], which is clearly at odds with the 0.5–8% frequency reported in civilian TBI cases [10–15], and the 31–47% suffering subclinical TON [20–22], given the annual incidence of TBI, which is 258 per 100,000 in Europe with more than 350,000 hospital admissions annually in the UK [43, 44]. One reason for the lack of studies may therefore be underdiagnosis of the condition by ophthalmologists. An additional reason may be timing. It is notable that the CRASH study recruited patients within 8 h of TBI for neuroprotective therapy, whilst the included studies recruited patients within days to weeks. Cell death processes are initiated rapidly after injury and most animal studies administer steroid treatment coincident with or very soon after injury [45, 46]. Given the apparent barriers to diagnosis of TON in routine clinical practice, diagnosis sufficiently quickly to recruit to a trial of an investigational medical product within hours of injury in patients who will usually be unconscious as a result of TBI would be possible, but may explain the paucity of studies. To improve the opportunities for research in this area, the first step will be to improve the likelihood of diagnosis in affected patients, which would require closer working with trauma specialists and may also drive greater consideration of systemic safety data.

The scientific rationale for neuroprotection is that some potentially functional RGC degenerate as a result of aberrant cell death signalling pathway activation and many studies have examined the potential to protect RGC with locally and systemically administered neuroprotective therapies, but most assess RGC survival as cell bodies in the retina, not in terms of intact or functional axonal connections to CNS targets, and animal models (and ultimately clinical diseases) in which the primary insult is to the axon therefore rely on axonal regeneration to demonstrate functional benefit [45–47]. Importantly the literature on axonal protection after TON is much more sparse than that on neuroprotection, particularly with respect to improved visual outcome, but one experimental study using the optic nerve crush model found greater axonal loss with methylprednisolone treatment [48]. The visual function benefits of neuroprotective or axon-protective interventions are rarely described [49]. There is a need for a greater pre-clinical focus on therapies to enhance functional axonal survival to develop new candidate translational treatments.

## Conclusion

There is no evidence of benefit for any pharmacologic or surgical intervention in treatment of TON. There is strong evidence of harm for treatment with megadose corticosteroids and surgical canal decompression.

## Summary

### What was known before

- Traumatic optic neuropathy is underdiagnosed and is variably treated in routine clinical practice.

### What this study adds

- Clinical trials to date have not provided clear benefits for therapeutic interventions.
- Megadose intravenous methylprednisolone is harmful.
- Orbital compartment syndrome requires urgent decompression.
